# Comparative Efficacy and Safety of Vonoprazan–Amoxicillin Dual Therapy Versus Clarithromycin-Based Standard Triple Therapy for *Helicobacter pylori* Eradication: A Systematic Review and Meta-Analysis of Randomized Controlled Trials

**DOI:** 10.3390/jcm15124647

**Published:** 2026-06-15

**Authors:** Nikolay Georgiev, Mihaela Malcheva, Plamen Penchev

**Affiliations:** 1 Department of Gastroenterology and Hepatology, Transport Hospital—Varna, 9000 Varna, Bulgaria; 2 Faculty of Medicine, Medical University of Plovdiv, 4001 Plovdiv, Bulgaria

**Keywords:** *Helicobacter pylori*, vonoprazan, amoxicillin, proton pump inhibitor, clarithromycin

## Abstract

**Introduction:** The declining efficacy of standard triple therapy for *Helicobacter pylori* (*H. pylori*) eradication, largely driven by increasing antibiotic resistance, has highlighted the need for alternative treatment strategies. Vonoprazan–amoxicillin dual therapy (VDT) has emerged as a promising regimen due to the potent and sustained acid suppression provided by vonoprazan. This meta-analysis aims to compare the efficacy and safety of VDT versus clarithromycin-based standard triple therapy (STT) for *H. pylori* eradication in adults. **Methods:** A systematic search of PubMed, Scopus, and the Cochrane Library was conducted from inception to 15 March 2026 for randomized controlled trials (RCTs) comparing VDT (vonoprazan plus amoxicillin) with STT (proton pump inhibitor, amoxicillin, and clarithromycin) for *H. pylori* eradication (PROSPERO “CRD420261357715”). Heterogeneity was assessed using the I^2^ statistic and Cochran’s Q test. Risk ratios (RRs) with 95% confidence intervals (CIs) were calculated using the Mantel–Haenszel method within a restricted maximum-likelihood random-effects model. **Results:** Five RCTs were included with 1363 patients (VDT: 680, STT: 683). VDT demonstrated a significantly higher *H. pylori* eradication rate compared with STT (RR 1.17; 95% CI [1.07; 1.27]; *p* = 0.007; I^2^ = 11%). **Conclusions:** This meta-analysis suggests that VDT may be associated with higher *H. pylori* eradication rates than clarithromycin-based STT. Further large, well-designed RCTs are needed before firm first-line recommendations can be made.

## 1. Introduction

*Helicobacter pylori (H. pylori)* infection remains one of the most prevalent chronic bacterial infections worldwide, affecting nearly half of the global population and representing a major public health concern [1,2]. It is strongly associated with chronic gastritis, peptic ulcer disease, mucosa-associated lymphoid tissue lymphoma, and gastric adenocarcinoma, the latter being one of the leading causes of cancer-related mortality globally [2,3]. Consequently, effective eradication therapy is essential not only for symptom relief but also for the prevention of long-term complications, including gastric malignancy [2].

For decades, standard triple therapy consisting of a proton pump inhibitor (PPI) combined with amoxicillin and clarithromycin or metronidazole has been widely used as first-line treatment. However, the efficacy of this regimen has progressively declined, largely due to the increasing prevalence of antibiotic resistance, particularly to clarithromycin and metronidazole. In many regions, eradication rates with standard triple therapy have fallen below the acceptable threshold of 80%, raising concerns regarding its continued empirical use [2,4].

Vonoprazan, a novel potassium-competitive acid blocker (P-CAB), has emerged as a promising alternative acid-suppressive agent. Compared with conventional PPIs, vonoprazan provides more rapid, potent, and sustained gastric acid suppression, thereby potentially enhancing the bactericidal activity of amoxicillin, whose efficacy is pH-dependent [2]. This pharmacological advantage has led to increasing interest in vonoprazan–amoxicillin dual therapy as a simplified regimen that may reduce antibiotic exposure while maintaining or improving eradication success. Recent randomized controlled trials (RCTs) have reported encouraging findings, suggesting that dual therapy may achieve eradication rates comparable or superior to conventional triple therapy, with a potentially lower incidence of adverse effects [5,6].

Despite these promising data, the evidence remains inconclusive. Existing studies have reported heterogeneous results, likely influenced by differences in treatment duration, dosing regimens, regional resistance patterns, and study populations. Furthermore, although several previous meta-analyses have evaluated vonoprazan-based regimens, the comparative efficacy and safety of vonoprazan–amoxicillin dual therapy specifically versus standard triple therapy in RCTs has not been comprehensively synthesized, leaving a gap in the literature [7,8].

Although recent meta-analyses have evaluated vonoprazan-based regimens for *H. pylori* eradication, many have pooled heterogeneous treatment strategies, including vonoprazan triple therapy, bismuth-containing regimens, non-randomized studies, or comparators other than conventional clarithromycin-based PPI triple therapy [1,7]. The added value of the present study is its focused evaluation of vonoprazan–amoxicillin dual therapy (VDT) compared specifically with clarithromycin-based standard triple therapy (STT). This design was selected to provide a targeted estimate of the comparative efficacy of a simplified dual regimen that removes clarithromycin while preserving amoxicillin and potent acid suppression as the therapeutic core.

Therefore, the present systematic review and meta-analysis aimed to compare the efficacy and safety of VDT versus STT for *H. pylori* eradication in adults, using evidence exclusively from RCTs.

## 2. Methods

### 2.1. Eligibility Criteria

This systematic review and meta-analysis followed the Cochrane Handbook for Systematic Reviews of Interventions and the Preferred Reporting Items for Systematic Reviews and Meta-Analyses (PRISMA) statement. The completed PRISMA checklist is provided in Supplementary Materials [9,10]. This meta-analysis did not require Institutional Review Board approval because it used data from previously published and publicly available articles. Studies that met all the following criteria were included in the meta-analysis: (1) adult patients (≥18 years) with confirmed *H. pylori* infection diagnosed by urea breath test, stool antigen test, rapid urease test, histology, or culture; (2) intervention with vonoprazan–amoxicillin dual therapy; (3) comparator consisting of clarithromycin-based standard triple therapy, defined as a proton pump inhibitor plus amoxicillin plus clarithromycin; (4) reporting at least one outcome of interest, including *H. pylori* eradication rate confirmed by urea breath test, stool antigen test, or biopsy-based methods, and/or safety outcomes; and (5) randomized controlled trial design. Studies were excluded if they included pediatric populations (<18 years), patients without confirmed *H. pylori* infection, vonoprazan triple or quadruple therapy without a separate extractable VDT arm, non-clarithromycin-based comparator regimens, no standard triple therapy comparator, non-extractable outcomes, overlapping populations, case reports or case series, editorials, letters, conference abstracts without full-text availability, animal studies, or in vitro studies. The restrictive eligibility criteria were intentionally applied to maintain a clinically homogeneous comparison between VDT and clarithromycin-based STT. Studies were excluded if they evaluated vonoprazan triple therapy, bismuth quadruple therapy, non-clarithromycin-based comparators, non-randomized designs, overlapping populations, conference abstracts without full-text data, or outcomes that were not extractable for the comparison of interest. Consequently, only five RCTs fulfilled all eligibility criteria and were included in the final quantitative synthesis. This systematic review and meta-analysis was registered with the International Prospective Register of Systematic Reviews (PROSPERO) under the ID “CRD420261357715”.

### 2.2. Search Strategy and Data Extraction

We systematically searched PubMed, Scopus, and Cochrane Central from inception to 15 March 2026 with the following search strategy: (“*Helicobacter pylori*” [Mesh] OR “H. pylori” OR “*Helicobacter pylori*”) AND (vonoprazan OR “potassium-competitive acid blocker” OR P-CAB) AND (“dual therapy” OR (vonoprazan AND amoxicillin) AND (“triple therapy” OR “standard therapy” OR “clarithromycin-based therapy” OR amoxicillin OR clarithromycin) AND (randomized controlled trial[pt] OR randomized[tiab] OR randomised[tiab]). Restrictions were applied to only English-language articles. Gray literature was excluded. We manually searched the references of all included studies to identify any additional studies. Two authors (N.G. and M.M.) independently extracted the data using predefined search criteria, quality assessment methods, and Rayyan software https://www.rayyan.com/ (20 April 2026) [11]. Any disagreements between these authors were resolved through consensus. When available, antimicrobial susceptibility data were extracted, including clarithromycin and amoxicillin resistance status, resistance testing method, and eradication rates stratified by resistance status. However, a formal pooled resistance-stratified analysis was planned only if at least two studies reported extractable arm-specific eradication outcomes according to antimicrobial resistance status. For efficacy outcomes, we extracted the primary eradication dataset reported by each original RCT, prioritizing the full-analysis or modified intention-to-treat population when available. If a full-analysis or modified intention-to-treat population was not reported, the available-case efficacy population used in the original study was extracted. Per-protocol data were not used as the primary dataset unless no other efficacy population was available.

### 2.3. Endpoints and Subgroup Analyses

The primary outcome was *H. pylori* eradication. Safety outcomes were extracted as reported in the original studies; however, adverse events were summarized narratively because safety endpoints were not reported consistently enough for quantitative pooling. For eradication rates, we additionally conducted a subgroup analysis based on the geographic region, and risk of bias.

### 2.4. Quality Assessment

The risk of bias was assessed using the Cochrane Collaboration’s tool for assessing the risk of bias in randomized studies of interventions (RoB2) [12]. The RoB2 tool categorizes the risk of bias as low, some concerns, or high. Two authors (N.G. and P.P.) independently performed the assessments, resolving disagreements through consensus. Publication bias was evaluated using contour-enhanced funnel plots with the trim-and-fill method, which allows for a better interpretation of asymmetry related to statistical significance thresholds. Additional methods, such as p-curve or p-uniform analysis, were not feasible due to the absence of reported exact *p*-values or test statistics in all included studies. Following the Cochrane guidelines, the Egger test was not performed because fewer than 10 studies were included in the meta-analysis [9].

### 2.5. Statistical Analysis

The same predefined extraction hierarchy was applied consistently across all studies for the primary eradication analysis to avoid mixing randomized, per-protocol, and safety populations within the same pooled estimate. Risk ratios (RR) with 95% confidence intervals (CI) were computed to compare effects for binary endpoints using the Mantel-Haenszel method [13]. The restricted maximum-likelihood estimator random-effects model was applied for binary data to account for heterogeneity and small sample sizes [14,15]. Heterogeneity was assessed using the I^2^ statistic and the Cochran’s Q test. Two-sided *p*-values < 0.05 were regarded as statistically significant. Clinical heterogeneity was anticipated because the included RCTs differed in treatment duration, amoxicillin dosing frequency, PPI comparator, and geographic region. Nevertheless, pooling was considered appropriate because all studies addressed the same focused clinical comparison: vonoprazan–amoxicillin dual therapy versus clarithromycin-based PPI standard triple therapy for *H. pylori* eradication. Safety outcomes were extracted as reported in the original studies; however, adverse events were summarized narratively because safety endpoints were not reported consistently enough for quantitative pooling. Heterogeneity was further explored using prespecified subgroup analyses based on geographic region and risk of bias, leave-one-out sensitivity analyses, and Baujat plots. Meta-regression was not performed because only five RCTs were included. With fewer than 10 studies, meta-regression is underpowered, produces unstable estimates, and may generate misleading associations due to sparse study-level data. Therefore, the potential influence of treatment duration, amoxicillin dosing, PPI type, and geographic region was assessed descriptively and, where feasible, through subgroup and sensitivity analyses rather than formal meta-regression. Statistical analysis was performed using R software version 4.3.1 with the packages “metafor” and “meta” [16].

## 3. Results

### 3.1. Study Selection and Baseline Characteristics

**Figure 1:**
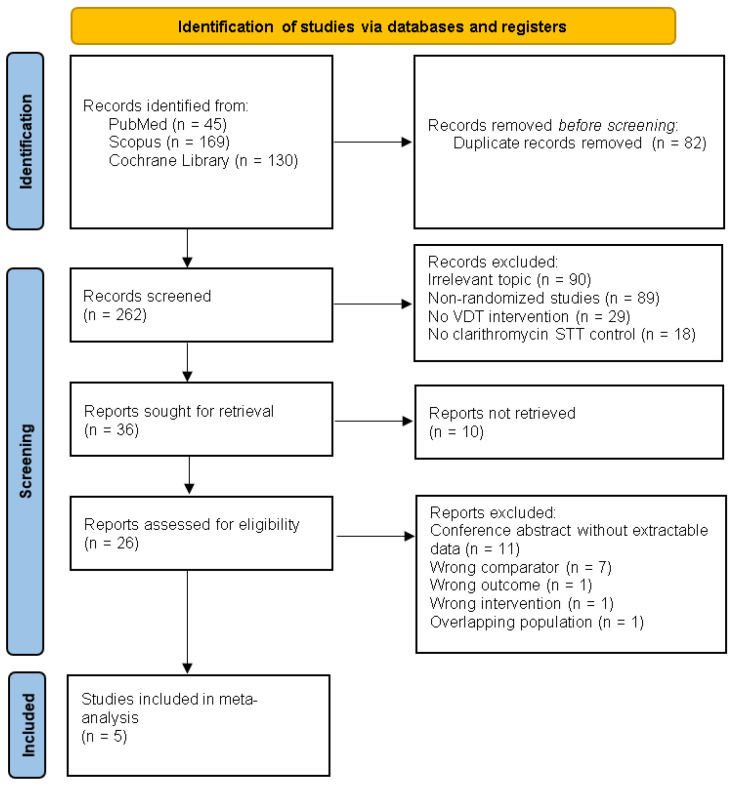
PRISMA flow chart and study selection.

The search identified 344 records. After removal of 82 duplicates, 262 records were screened by title and abstract. Of these, 226 were excluded: irrelevant topic (*n* = 90), non-randomized studies (*n* = 89), no VDT intervention (*n* = 29), and no clarithromycin-based STT comparator (*n* = 18). In total, 36 reports were sought for retrieval, of which 10 could not be retrieved. The remaining 26 full-text reports were assessed for eligibility. Twenty-one reports were excluded because of conference abstracts without extractable data (*n* = 11), wrong comparator (*n* = 7), wrong outcome (*n* = 1), wrong intervention (*n* = 1), or overlapping population (*n* = 1). Finally, five RCTs met the inclusion criteria and were included in the quantitative synthesis [17,18,19,20,21] (Figure 1). Because the included trials reported different analysis populations, randomized sample sizes, efficacy-analysis denominators, and safety-analysis denominators were extracted separately. For the primary eradication analysis, 1363 patients were included, with 680 in the VDT group and 683 in the STT group. The limited number of included RCTs reflects the intentionally narrow inclusion criteria requiring a direct randomized comparison between VDT and clarithromycin-based STT with extractable eradication and/or safety outcomes. The mean age of the VDT patients was 40 ± SD years, and of the STT group 39 ± SD years. Study characteristics are presented in Table 1. Antimicrobial susceptibility data were inconsistently reported across the included RCTs. Chey et al. performed culture and antimicrobial susceptibility testing and reported eradication outcomes according to clarithromycin-resistant and non-resistant strains. In contrast, the remaining included studies did not provide sufficient arm-specific resistance-stratified eradication data for quantitative synthesis. Therefore, a pooled analysis according to clarithromycin resistance status was not feasible. The included RCTs showed clinical variability in treatment duration, amoxicillin dosing frequency, PPI comparator, and geographic region; these variables were therefore considered when interpreting pooled estimates and explored qualitatively or through subgroup/sensitivity analyses where possible.

Differences between randomized sample sizes and efficacy-analysis denominators occurred because several trials excluded patients from the efficacy analysis because of missing confirmation of infection, missing test-of-cure data, loss to follow-up, or predefined analysis-population criteria. Therefore, the corrected analysis used efficacy denominators rather than safety denominators or baseline randomized denominators.

### 3.2. Pooled Analyses of the Included Studies

#### 3.2.1. *H. pylori* Eradication Rates

**Figure 2:**
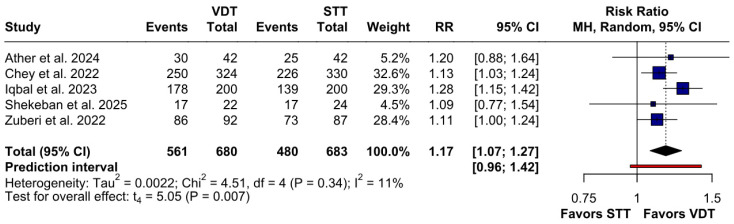
Forest plot comparing *H. pylori* eradication rates between VDT and clarithromycin-based STT [17,18,19,20,21].

**Figure 3:**
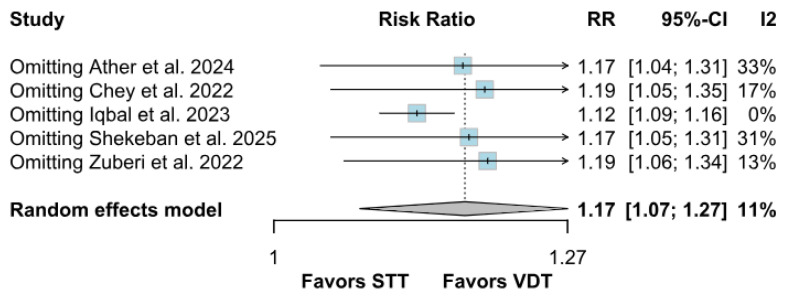
In terms of eradication rates, the overall effect size remained consistent across all iterations and the result remained significant in all cases. Heterogeneity remained low throughout all iterations [17,18,19,20,21].

**Figure 4:**
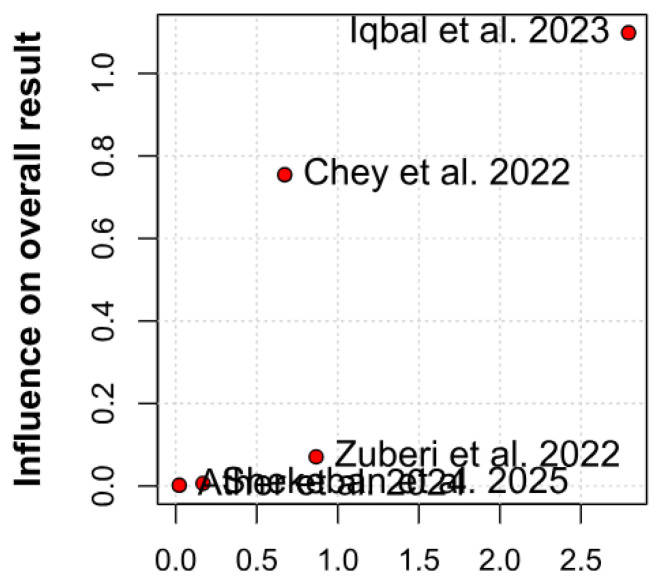
Baujat plot. The study by Iqbal et al. 2023 was discovered as potentially influential, contributing substantially to the overall result and heterogeneity [17,18,19,20,21].

Table 2 shows the primary eradication analysis used the predefined efficacy-analysis hierarchy described above; therefore, the pooled estimate was based on the main efficacy population reported by each trial rather than safety denominators or per-protocol denominators. VDT demonstrated a significantly higher *H. pylori* eradication rate compared with STT (RR 1.17; 95% CI [1.07; 1.27]; *p* = 0.007; I^2^ = 11%) (Figure 2). The LOO analysis showed that the pooled effect estimate remained significant after sequential exclusion of each individual study, suggesting that the overall result was not driven by any single trial (RR 1.17; 95% CI [1.07; 1.27]; *p* = 0.007; I^2^ = 11%) (Figure 3). The Baujat plot identified the study by Iqbal et al. 2023 [19] as potentially influential, contributing substantially to the overall result and heterogeneity (Figure 4).

#### 3.2.2. Adverse Effects

A quantitative meta-analysis of adverse events was not performed because safety outcomes were reported inconsistently across the included RCTs. Some studies reported treatment-emergent adverse events at the patient level, whereas others reported selected adverse-event categories, severity-based events, or total numbers of events. Because these outcomes were not directly comparable and category-specific events may overlap within the same patient, pooling them as “any adverse event” could produce misleading estimates. Overall, the available safety data did not suggest a clear safety disadvantage of VDT compared with STT; however, firm comparative safety conclusions cannot be drawn from the current evidence.

### 3.3. Subgroup Analyses

#### 3.3.1. Risk of Bias (ROB)

**Figure 5:**
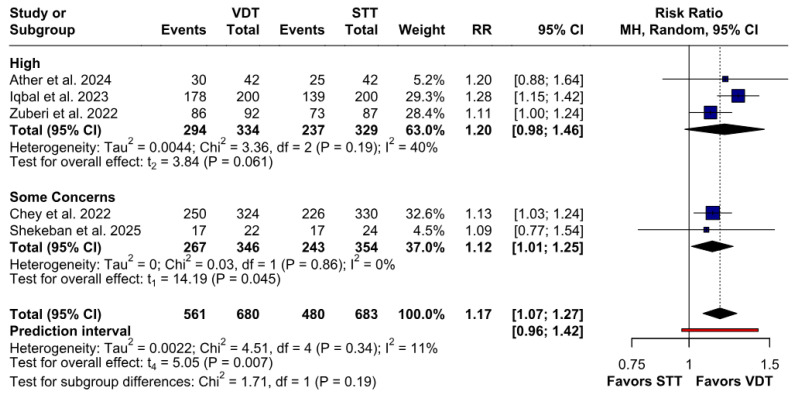
Subgroup analysis for eradication rates stratified by ROB [17,18,19,20,21].

Subgroup analysis by ROB showed that VDT numerically favored higher eradication rates in both subgroups. In studies judged as high risk of bias, the effect did not reach statistical significance (RR 1.20; 95% CI [0.98; 1.46]; I^2^ = 40%). In studies with some concerns, VDT was associated with higher eradication rates (RR 1.12; 95% CI [1.01; 1.25]; I^2^ = 0%). No statistically significant subgroup difference was observed by ROB category (*p* = 0.19) (Figure 5).

#### 3.3.2. Geographic Region

**Figure 6:**
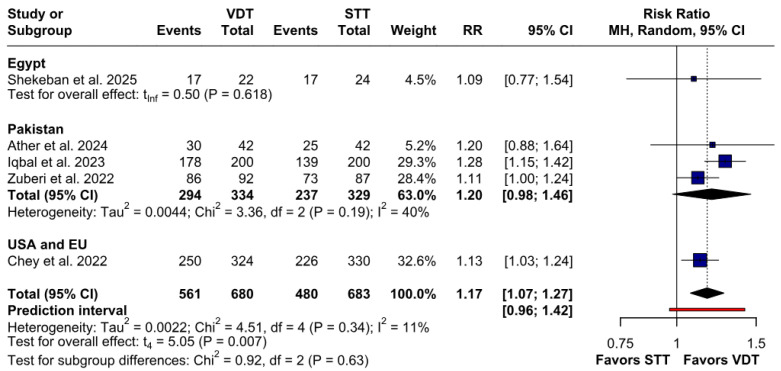
Subgroup analysis for eradication rates stratified by country [17,18,19,20,21].

Subgroup analysis by geographical region showed that VDT numerically favored higher *H. pylori* eradication rates across all regions. The effect was not statistically significant in Egypt (RR 1.09; 95% CI [0.77; 1.54]; *p* = 0.618) or Pakistan (RR 1.20; 95% CI [0.98; 1.46]; I^2^ = 40%), but was significant in the USA/EU subgroup (RR 1.13; 95% CI [1.03; 1.24]). Overall, VDT was associated with higher eradication rates than STT (RR 1.17; 95% CI [1.07; 1.27]; *p* = 0.007; I^2^ = 11%). No significant subgroup difference was observed by region (*p* = 0.63) (Figure 6).

### 3.4. Quality Assessment

**Figure 7:**
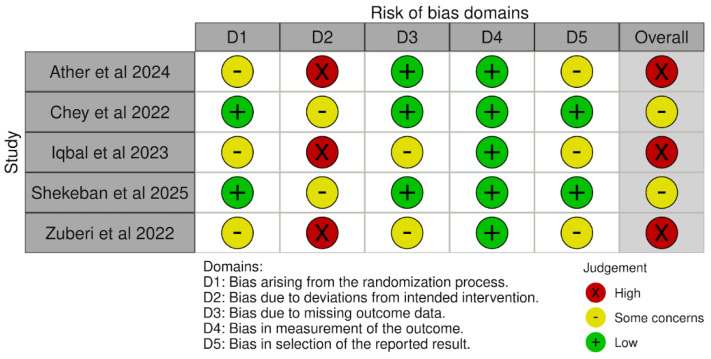
ROB assessment in the RCTs [17,18,19,20,21].

**Figure 8:**
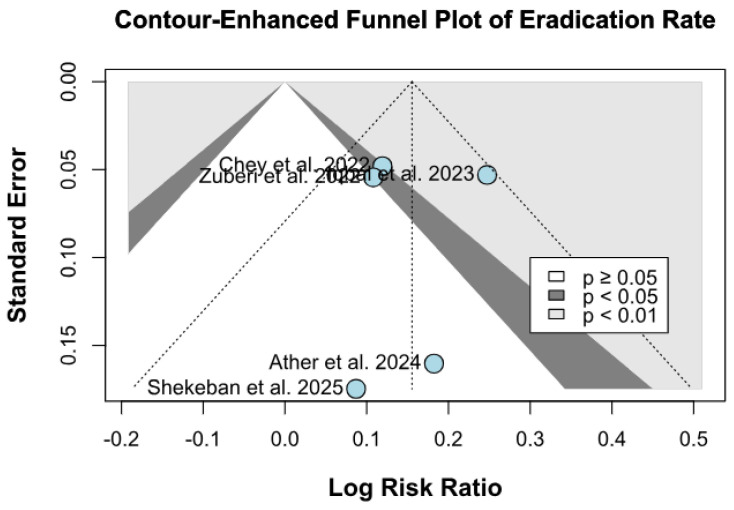
Contour-enhanced trim-and-fill funnel plot for *H. pylori* eradication. The plot illustrates individual study weights against point estimates [17,18,19,20,21].

Among the five included RCTs, two were assessed as having some concerns risk of bias, and three as having a high risk of bias based on the RoB2 tool. The detailed evaluation is presented in Figure 7. Publication bias was evaluated using contour-enhanced trim-and-fill funnel-plot analyses, plotting individual study weights against point estimates. The funnel plots for the outcome did show some asymmetry, but given the small number of included studies, visual interpretation is limited (Figure 8).

## 4. Discussion

The present systematic review and meta-analysis of five RCTs including 1363 patients in the primary eradication analysis suggests that VDT may be associated with higher *H. pylori* eradication rates than clarithromycin-based STT. Specifically, VDT was associated with a 17% relative increase in eradication success (RR 1.17, 95% CI 1.07–1.27), with low heterogeneity (I^2^ = 11%), suggesting a consistent treatment effect across studies. From a clinical perspective, these findings suggest that VDT may be a promising alternative to clarithromycin-based STT, particularly in settings where clarithromycin resistance or prior macrolide exposure reduces the expected efficacy of STT. However, given the limited number of RCTs, protocol-level heterogeneity, geographic concentration of the evidence, and risk-of-bias concerns, the current evidence should be considered suggestive rather than definitive [17,18,19,20,21].

The included trials differed in treatment duration, amoxicillin dose and dosing frequency, PPI comparator, and geographic region. These differences represent important sources of clinical heterogeneity and may influence eradication rates. However, all included studies addressed the same clinically relevant comparison between VDT and clarithromycin-based STT, which justified quantitative synthesis using a random-effects model. The low statistical heterogeneity observed for the primary eradication endpoint suggests that, despite protocol-level differences, the direction of effect was reasonably consistent across studies. Nevertheless, given the small number of RCTs, the pooled estimate should be interpreted cautiously and considered an average effect across related but not identical therapeutic protocols.

The superior efficacy of VDT is biologically plausible and largely attributable to the pharmacological advantages of vonoprazan. Unlike conventional proton pump inhibitors (PPIs), vonoprazan is a potassium-competitive acid blocker (P-CAB) that provides rapid, potent, and sustained gastric acid suppression from the first administered dose [5]. This is particularly important because the antimicrobial efficacy of amoxicillin against *H. pylori* is strongly pH-dependent. Higher intragastric pH promotes bacterial replication, rendering the microorganism more susceptible to cell wall-active agents such as amoxicillin [2,5]. Murakami et al. first demonstrated the clinical superiority of vonoprazan-containing regimens in a phase III randomized trial, establishing the pharmacodynamic rationale that later supported the development of dual therapy protocols [5].

Our findings are highly consistent with previous systematic reviews and meta-analyses. Feng et al. reported that vonoprazan–amoxicillin dual therapy achieved excellent eradication outcomes with a favorable safety profile [1]. Similarly, Zhou et al. demonstrated that vonoprazan-based dual therapy was significantly superior to PPI-based regimens in randomized trials [22]. More recently, Zhang et al. confirmed these findings in a dedicated meta-analysis, reporting high eradication rates and no excess adverse events [23]. A network meta-analysis by Liu et al. further supported the efficacy of VDT and suggested that this regimen may rank among the most effective first-line therapies currently available [24]. The concordance between our pooled results and these prior analyses supports the consistency of the observed efficacy signal, although the certainty of evidence remains limited by the small number of RCTs and risk-of-bias concerns [25].

A possible explanation for the relative advantage of VDT over clarithromycin-based STT is the declining effectiveness of clarithromycin-containing regimens in settings with increasing clarithromycin resistance [2]. However, this mechanism could not be directly tested in the present meta-analysis because antimicrobial susceptibility data were not consistently reported across the included RCTs. Among the included studies, only Chey et al. provided extractable eradication outcomes stratified by clarithromycin resistance status, while the remaining trials did not report sufficient arm-specific resistance-stratified data [18]. Therefore, the relationship between clarithromycin resistance and the observed pooled eradication effect should be interpreted as biologically plausible and supported by external evidence, rather than as a finding directly demonstrated by our meta-analysis.

Although VDT avoids clarithromycin exposure and may therefore be considered an antibiotic-sparing regimen, this study did not directly evaluate microbiological outcomes, changes in antimicrobial resistance, gut microbiome effects, or population-level consequences of antibiotic use. Accordingly, any potential antibiotic-stewardship implication should be interpreted as hypothesis-generating rather than directly demonstrated by the present meta-analysis.

Subgroup analyses by geographic region suggested that the direction of effect generally favored VDT across the included regions. However, these findings should be interpreted cautiously because each subgroup contained few studies, and regional antimicrobial resistance patterns were not consistently available [2,22]. Although no statistically significant subgroup interaction was observed, the current evidence is insufficient to confirm that the relative benefit of VDT is generalizable across all healthcare settings. Further multinational RCTs with standardized resistance testing are needed.

Safety outcomes were reported heterogeneously across the included RCTs, preventing a reliable pooled meta-analysis of adverse events. Some studies reported treatment-emergent adverse events at the patient level, whereas others reported selected symptom categories, severity-based events, or total event counts, which may overlap within the same patient. Therefore, firm comparative safety conclusions cannot be drawn. Overall, the available data did not suggest a clear safety disadvantage of VDT, but future RCTs should use standardized definitions and separately report any adverse event, treatment-related adverse events, serious adverse events, and discontinuations due to adverse events.

The robustness of our findings is supported by the LOO sensitivity analyses, which showed that the pooled effect estimate remained stable after sequential exclusion of each individual study. This suggests that no single trial disproportionately influenced the overall result. Furthermore, the low heterogeneity observed for the primary efficacy endpoint enhances confidence in the reliability of the pooled estimate. Although the Baujat analysis identified Iqbal et al. as a potentially influential study, its removal did not materially alter the significance or direction of the results.

From a clinical perspective, these findings suggest that VDT may be a promising alternative to clarithromycin-based STT, particularly in settings with high clarithromycin resistance or prior macrolide exposure. However, given the limited number of RCTs, protocol-level heterogeneity, geographic concentration of the evidence, and risk-of-bias concerns, the current evidence should be considered suggestive rather than definitive.

The global clinical applicability of VDT also depends on local availability, regulatory approval, cost, and reimbursement of vonoprazan. Although VDT may be a promising alternative to clarithromycin-based STT in settings where vonoprazan is accessible, its implementation may be limited in countries where vonoprazan is unavailable, not reimbursed, or substantially more expensive than conventional PPI-based regimens. Therefore, the present findings are most directly applicable to healthcare systems in which vonoprazan is available and where clarithromycin resistance or prior macrolide exposure reduces the expected effectiveness of conventional STT. In regions without access to vonoprazan, these results should be interpreted as evidence supporting further evaluation rather than immediate global implementation.

Several limitations should be acknowledged. First, only five RCTs were included, limiting the statistical power for subgroup and publication bias analyses. Second, three studies were classified as having a high risk of bias, which may affect the certainty of evidence. Third, the included RCTs differed in treatment duration, amoxicillin dosing frequency, PPI comparator, and geographic region. Although random-effects modeling and sensitivity analyses were used to address this variability, the small number of studies prevented formal meta-regression. Fourth, long-term recurrence and reinfection outcomes were not consistently reported. Fifth, antimicrobial susceptibility data, particularly clarithromycin resistance status, were not consistently available across the included RCTs, preventing resistance-stratified subgroup analysis or meta-regression. Sixth, safety outcomes were underpowered and inconsistently reported, preventing a reliable pooled analysis of overall or category-specific adverse events. In addition, this meta-analysis did not assess microbiological outcomes, changes in antimicrobial resistance, gut microbiome effects, or other population-level consequences of antibiotic use; therefore, potential antibiotic-stewardship implications remain speculative. Finally, the variable global availability, regulatory approval, and cost of vonoprazan may limit the generalizability and immediate clinical applicability of VDT in many healthcare systems.

## 5. Conclusions

This systematic review and meta-analysis, including 1363 patients in the primary eradication analysis, suggests that VDT may be associated with higher *H. pylori* eradication rates than clarithromycin-based STT. However, the certainty of evidence is limited by the small number of RCTs, risk-of-bias concerns, protocol-level heterogeneity, inconsistent adverse-event reporting, and incomplete antimicrobial resistance data. Further large, well-designed multicenter RCTs and cost-effectiveness studies in diverse healthcare settings are needed before firm first-line recommendations and broad global implementation can be made.

## Figures and Tables

**Table 1:** Baseline characteristics of the included studies.

Characteristics/Study	Ather et al. 2024 [17]	Chey et al. 2022 [18]	Iqbal et al. 2023 [19]	Shekeban et al. 2025 [20]	Zuberi et al. 2022 [21]
Study design	RCT	RCT	RCT	RCT	RCT
Country/region	Pakistan	USA and Europe	Pakistan	Egypt	Pakistan
No. patients	84	654	400	46	179
VDT, *n*	42	324	200	22	92
STT, *n*	42	330	200	24	87
Age *	VDT: 39.12 STT: 38.51	VDT: 51.8 STT: 51.8	VDT: 37.78 STT: 32.23	VDT: 31 STT: 35.5	VDT: 41.4 STT: 40.2
Males, %	VDT: 71 STT: 66	VDT: 39 STT: 38	VDT: 43 STT: 39	VDT: 29 STT: 27	VDT: 55 STT: 54
Treatment duration	14 days	14 days	4 weeks	14 days	14 days
VDT regimen	V 20 mg BID + A 1 g BID	V 20 mg BID + A 1 g TID	V 20 mg BID + A 1 g every 8 h	V 20 mg BID + A/clav 875/125 mg TID	V 20 mg BID + A 1 g BID
STT regimen	O 20 mg BID + A 1 g BID + C 500 mg BID	L 30 mg BID + A 1 g BID + C 500 mg BID	L 20 mg BID + A 1 g BID + C 500 mg BID	PPI standard/double dose + A/clav 875/125 mg BID + C 500 mg BID	O 20 mg BID + A 1 g BID + C 500 mg BID
Diagnostic method	C13 UBT and Campylobacter test reported; stool antigen also reported	13C-UBT plus histology/culture confirmation for efficacy analyses	Stool antigen test	Stool antigen test	Stool antigen or histopathology
Eradication confirmation method/timing	Stool antigen test, 4 weeks after therapy	13C-UBT at week 6, approximately 4 weeks after therapy	Stool antigen test after therapy	Stool antigen test ≥1 month after treatment completion	Stool antigen test, 4 weeks after therapy
Primary analysis population used	Available-case efficacy population	Full-analysis set	Reported randomized/analyzed population	mITT	Available-case efficacy population
Prior antibiotic exposure	Previous *H. pylori* eradication therapy excluded	Treatment-naïve patients	Recent antibiotics excluded	Previous eradication therapy and recent antimicrobials excluded	Recent antibiotics excluded
Antimicrobial resistance data	NR	Culture and antimicrobial susceptibility testing performed; eradication reported by clarithromycin resistance status	NR	Background/regional resistance discussed; no extractable arm-specific susceptibility-stratified eradication data	NR
Regional resistance information	Clarithromycin resistance discussed in background only	USA/Europe; clarithromycin-resistant strains reported	Resistance discussed in background only	Regional resistance patterns discussed, including limited/controversial Egyptian data	Clarithromycin resistance discussed in background only
Blinding	NR	VDT open-label; triple therapy double-blind	NR	Open-label	NR

Abbreviations: VDT, vonoprazan–amoxicillin dual therapy; STT, standard triple therapy; V, vonoprazan; A, amoxicillin; A/clav, amoxicillin/clavulanate; C, clarithromycin; O, omeprazole; L, lansoprazole; PPI, proton pump inhibitor; BID, twice daily; TID, three times daily; UBT, urea breath test; mITT, modified intention-to-treat; NR—not reported * Age is presented as reported in the original studies.

**Table 2:** Population data information.

Study	Population Used
Ather et al. [17]	Available-case efficacy population
Chey et al. [18]	Full-analysis set
Iqbal et al. [19]	Reported randomized/analyzed population
Shekeban et al. [20]	Modified intention-to-treat
Zuberi et al. [21]	Available-case efficacy population

## Data Availability

No new data were created or analyzed in this study. Data sharing is not applicable to this article.

## Supplementary Materials

The following supporting information can be downloaded at: https://www.mdpi.com/article/10.3390/jcm15124647/s1, PRISMA cheklist.
